# Social acceptability and perceived impact of a community-led cash transfer programme in Zimbabwe

**DOI:** 10.1186/1471-2458-13-342

**Published:** 2013-04-15

**Authors:** Morten Skovdal, Phyllis Mushati, Laura Robertson, Shungu Munyati, Lorraine Sherr, Constance Nyamukapa, Simon Gregson

**Affiliations:** 1 Department of Health Promotion and Development, University of Bergen, Bergen, Norway; 2 Biomedical Research and Training Institute, Harare, Zimbabwe; 3 School of Public Health, Imperial College London, London, UK; 4 Department of Infection and Population Health, University College London, London, UK

**Keywords:** Cash transfers, Social protection, Incentives, Child health, Community participation HIV/AIDS, Zimbabwe

## Abstract

**Background:**

Cash transfer programmes are increasingly recognised as promising and scalable interventions that can promote the health and development of children. However, concerns have been raised about the potential for cash transfers to contribute to social division, jealousy and conflict at a community level. Against this background, and in our interest to promote community participation in cash transfer programmes, we examine local perceptions of a community-led cash transfer programme in Eastern Zimbabwe.

**Methods:**

We collected and analysed data from 35 individual interviews and three focus group discussions, involving 24 key informants (community committee members and programme implementers), 24 cash transfer beneficiaries, of which four were youth, and 14 non-beneficiaries. Transcripts were subjected to thematic analysis and coding to generate concepts.

**Results:**

Study participants described the programme as participatory, fair and transparent – reducing the likelihood of jealousy. The programme was perceived to have had a substantial impact on children’s health and education, primarily through aiding parents and guardians to better cater for their children’s needs. Moreover, participants alluded to the potential of the programme to facilitate more transformational change, for example by enabling families to invest money in assets and income generating activities and by promoting a community-wide sense of responsibility for the support of orphaned and vulnerable children.

**Conclusion:**

Community participation, combined with the perceived impact of the cash transfer programme, led community members to speak enthusiastically about the programme. We conclude that community-led cash transfer programmes have the potential to open up for possibilities of participation and community agency that enable social acceptability and limit social divisiveness.

## Background

Cash transfers are increasingly used in sub-Saharan Africa as a social protection instrument to address poverty and improve the health and well-being of children living in poor resource and high HIV prevalence areas. Small amounts of cash given to poor households on a regular and predictable (often monthly or bi-monthly) basis allow for control, independence and decision making [1,2]. However, cash transfers, which target some households and not others, can misfire and lead to conflict, jealousy and other unintended consequences. It is against this background that we explore community perceptions of a community-led cash transfer programme in Zimbabwe and discuss the role of community participation in contributing to the acceptability of cash transfers, reducing the risk of unintended consequences, and meeting programme objectives.

Conditional cash transfers gained popularity in South America where long-standing programmes have demonstrated their success through significant improvements in access to education, food security, child health and growth [2-6]. In addition to highlighting significant health impacts, studies from the region have also indicated the potential of cash transfers to undermine local coping strategies [7] as well as reinforcing gender roles and responsibilities in managing the impact of poverty [8-11].

Nonetheless, inspired by the potential of cash transfers in South America, in 2006, the African Union spearheaded ‘The Livingstone Call for Action’, which brought together Ministers and senior government officials from 13 African countries to discuss the role of cash transfers. The meeting firmly established cash transfers as a viable and promising social protection strategy for Africa. As such, a number of sub-Saharan African countries have begun to design, implement and scale-up cash transfer programmes (see for examples, [12,13]).

The early experiences of unconditional cash transfer programmes in sub-Saharan Africa have been succinctly discussed by Adato and Bassett [14] in their review of published evaluations. They found several positive effects of unconditional cash transfers, including improved nutritional status of children (Malawi, South Africa and Zambia), reduced reports of illness amongst children (Malawi and Zambia) and increased school enrolment and attendance (Ethiopia, South Africa, Zambia and Malawi). More recently, a conditional cash transfer programme in Malawi observed significant reductions in risky sexual behaviour, early marriage and pregnancy amongst young women aged 13–22 years [15]. Our own study in Zimbabwe found cash transfers to i) increase school attendance amongst orphaned and vulnerable children; ii) increase birth registration of children in households receiving conditional cash transfers; and iii) to have no significant effect on vaccination uptake [16].

Although current evaluations of cash transfer programmes in sub-Saharan Africa indicate great potential, MacAusland and Riemenschneider [17] argue that the evaluation studies that dominate the cash transfer literature are too focused on ‘material’ and health gains, with less attention given to changes in the ‘relational’ and ‘symbolic’ dimensions that shape the social landscape in which cash transfer programmes are located. Social studies have sought to fill this gap by raising questions about the socio-ethical implications of cash transfers. For example, studies in Africa have reported on conflict and jealousy arising from the divisiveness of some households being targeted whilst others are not, even though they are all considered poor [17,18]. An in-depth study of female cash transfer recipients in South Africa found that although cash transfers provided women with a valuable safety net, helping them to cope with poverty and domestic obligations [19], these women also felt labelled as poor, stigmatised as lazy, and experienced shame through their association a cash transfer programme transfer [20]. Similar observations have been made in Kenya where cash transfer recipients deliberately kept their status as cash transfer recipients a secret out of fear of public opinion [21]. Such studies suggest that cash transfer programmes often fail to adequately resonate with local norms and structures, but provide us with few clues as to how we can create a better fit between local norms and structures and the very nature of cash transfers (i.e. some households receive a regular income whilst others do not) to achieve more widespread social acceptability – buy-in from community members – and in the process overcome potential unintended consequences.

In this paper we use qualitative data to explore community member’s experiences of a community-led cash transfer programme in Manicaland, eastern Zimbabwe. More specifically, we examine their perspectives on how participatory the programme was and how this in turn, combined with their perceived impact of the programme, helped achieve social acceptability. We hope that our partial focus on the implementation process, and lessons learned from embedding the cash transfer programme into a community context, provides useful insights to how cash transfer programmes can pay more attention to possibilities of participation and community agency, and thereby be more aligned with local realities, achieve social acceptability and meet programme objectives.

## Methods

This qualitative study forms part of a larger cluster-randomised trial of a cash transfer programme in eastern Zimbabwe. Ethical approval for the trial and this study was granted by the Imperial College Research Ethics Committee (ICREC_9_3_10), the Biomedical Research and Training Institute’s Institutional Review Board (AP81/09), and the Medical Research Council of Zimbabwe (MRCZ/A/1518). Informed and written consent was obtained from all participants upon the agreement that confidentiality would be ensured. We have therefore used pseudonyms throughout.

### Study location and the cash transfer programme

Zimbabwe has, over the past decade, experienced one of the world’s most severe HIV epidemics and a period of rapid economic decline. The combination of these two factors has led to a dramatic increase in the number of orphaned and other vulnerable children. Although Zimbabwe has seen a decline in HIV prevalence since the late 1990s (e.g., from 29.3% in 1997 to 15.6% in 2007) – fuelled by declines in risk behaviours and partner reductions [22,23] – a large number of people continue to experience the devastating effects of poverty and HIV. It is estimated that, with around 1.6 million children in Zimbabwe having lost one or both parents due to HIV and other causes, one out of four children, and the homes in which they are living, are in need of social protection [24]. In Manicaland Province where this study takes place, our own surveys indicate that 20.8% of children (data collected 2003/05) are orphaned [25,26]. Responding to the social protection needs of children in Zimbabwe, the Department of Social Services developed *The National Action Plan for Orphans and Vulnerable Children Phase II, 2011–2015*, prioritising cash transfers as a key strategy for the social protection of orphaned and vulnerable children.

The community-randomised cash transfer trial that we report on in this paper began in July 2009 across 30 communities in Manicaland. The programme was community-led and directed. Its design was informed by findings from a feasibility study conducted with a consultancy (Development Data), which asked local people and stakeholders about the desirability of a cash transfer programme and possible design features. Drawing on recommendations from the feasibility study, key entry points and implementation mechanisms through which the cash transfer programme could provide opportunities for community participation were identified. As a result, and through consultation with local leadership (village heads), it was agreed that the best way forward was to administer the programme through community-based Cash Transfer Committees (CTCs). To establish these committees, each community was divided into five areas, or villages, and the person from each village getting most votes was elected to become a member of the local community committee. It was the responsibility of the committee members to mobilise local villages within the community, facilitate village meetings to discuss and verify who was eligible to benefit from the programme, facilitate parenting skills classes as well as assist with cash distributions and the verification of compliance with conditions. Cash disbursements were made at pay-points in central locations in each community and facilitated by CTC members.

The communities were assigned to one of three study groups: control, cash transfers, or conditional cash transfers. The conditions required for the conditional cash transfer group were obtaining birth certificates, keeping children up-to-date with vaccinations and attending a growth-monitoring clinic twice a year, keeping school attendance above 90% of days each month, and attending parenting-skills classes. Eligible households were identified through a two-stage process (see also [27]). First, data from our population-based household survey were used to generate lists of eligible households. Beneficiaries had to be in the poorest quintile at baseline, host one or more orphaned children, be child-headed or contain a chronically ill or disabled household member. The lists of eligible households (according to the household survey) were taken to the communities for discussion and verification. This process helped us to identify a total of 2,844 households in the 20 ‘cash transfer’ communities of which 1,525 received unconditional cash transfers and 1,319 received conditional cash transfers. There were a further 1,199 households in the 10 control communities. Between January 2011 and January 2012, the targeted households received bi-monthly grants of US$18 plus an extra US$4 per child living in the household (up to a maximum of three children). The cash transfer programme was funded by the Programme of Support for the Zimbabwe National Action Plan for OVC and implemented in a partnership between the Biomedical Research and Training Institute, Catholic Relief Services in Zimbabwe, and the Diocese of Mutare Community Care Program.

### Study participants and sampling

This evaluation reports on the perspectives of 58 adults and 4 youth (aged 14–21 years) who participated in 35 structured interviews and three focus group discussions. To gather a broad range of perspectives, community members with varying degrees of involvement were invited to participate in the study. As detailed in Table 1, the study participants included 24 key informants (community committee members and programme implementers), 24 direct beneficiaries of the conditional (5 adults and 3 youth) and unconditional (15 adults and 1 youth) cash transfer arms and 14 non-beneficiaries. Participants were randomly selected from a list of programme stakeholders and recruited by Shona-speaking researchers from the Biomedical Research and Training Institute in consultation with community guides.

**Table 1:** Summary of study informants

	**Individual interviews**		**Focus groups**		**Total no. of interviews (participants)**
	**Adults**	**Youth**	**Adults**	**Youth**	
**Key Informants**	15	0	1 (9 part.)	0	16 (24 part.)
**Cash Transfer Beneficiaries**	6	1	1 (9 part.)	0	8 (16 part.)
**Conditional Cash Transfer Beneficiaries**	5	3	0	0	8 (8 part.)
**Non-beneficiaries**	5	0	1 (9 part.)	0	6 (14part.)
**Total no. of interviews (participants)**	31	4	3 (27 part.)	0	38 (62 part.)

### Data collection and analysis

All interviews, except for one interview that was conducted in English, were conducted in the local Shona language by experienced qualitative researchers. A topic guide was developed to explore the perspectives of beneficiaries, local stakeholders and community members at large. Topics covered by the interview guides included: how the programme was implemented, local understandings of the programme, cash spending, conditions, changes the programme had instigated, impact and local barriers to programme success. Youth were interviewed using the same topic guide, but by a research assistant with specialised social worker training pertaining to the challenges of doing research with children and youth (see for example [28]). The individual interviews lasted an average of 40 minutes, whilst the group interviews took an average of 94 minutes. The interviews were translated and transcribed into English and imported into a qualitative software package (Atlas.Ti) for coding and more in-depth examination. As we seek to report on more general perceptions of the programme, we did not aim to make links between participants from the three design groups (conditional cash transfer, unconditional cash transfer or control groups) and their individualised personal experiences, but rather to map the more general feeling of the programme as a whole. As such, the entire data corpus constitutes our unit of analysis rather than separate datasets for the different design groups.

The analysis involved a stage-wise process that was open for both *a priori* reasoning and surprises. The first step involved us reading and coding the transcripts. A total of 90 codes were generated from this process. However, as we do not seek to report on all the themes emerging from our qualitative analysis in this paper, but to explore community members’ perceptions of the programme, we only report on the 38 codes that have direct relevance to the topic of this paper (see Table 2). These codes were subsequently subjected to a thematic network analysis [29], involving the grouping together of codes into basic themes, which were subsequently grouped into higher order and more interpretative organising themes. This process, as well as analysing all the transcripts together, allowed us to map out some of the more prevalent experiences and perceptions as reported by the informants. As shown in Table 2, a total of six organising themes emerged from this analysis, giving us an insight to how community members in this context experienced a community-led cash transfer programme. We will now explore these six themes by systematically discussing the 14 basic themes emerging from our analysis.

**Table 2:** Global theme: Local perceptions of a community-led cash transfer programme

***Codes***	***Basic themes***	***Organising themes***
Community committees	Community members involved in the implementation	1. Community participation
Working with local leaders		
Selection process		
Drawing on local knowledge		
Compliance	Community members involved with the monitoring	
Formal monitoring		
Informal monitoring		
Buy-in and solidarity	Community-wide appreciation	2. Social acceptability
Community-wide benefits		
Cooperate with other services	Cash transfers complement other support services	
More holistic than other programmes		
BEAM		
Deserving beneficiaries	Fairness of the programme	
Transparency		
Access to uniforms	School attainment	3. Improved schooling and education
School attendance		
School over subscription		
Prompt payment of school fees		
School performance	School performance	
Food intake	Physical health	4. Improved health and well-being
Vaccination		
General health benefits		
Equality	Psychosocial health	
Reduced caregiver stress		
Understanding of children’s needs	Community wide awareness and response to children’s needs	5. Poverty reduction and social transformation
Community: agents of change		
Birth certificates obtained		
Empowered caregivers	Caregivers are empowered	
Platform for income generation		
Children are equal	Greater sense of equality and cohesion	
Enhanced community dialogue		
People are better off		
Narrow targeting	Jealousy	6. Persisting social and logistical challenges
Undeserving beneficiaries		
Illness and disability	Barriers to programme success	
Men taking the money		
Household dynamics		
Religion		

## Results

### Community participation

An integral part of the cash transfer programme was to mobilise community-based committees and enable them to lead the implementation process. This intrinsic recognition of having to involve community members in the programme implementation was generally appreciated. One community leader went so far as to say that the success of the programme was down to how “it valued people’s input” and “drew from the local way of doing things”. Community members were involved in different ways and at different levels. Village (Kraal) heads were consulted in the planning stages and for programme approval. Village heads were vital for the mobilisation of the communities and organising community meetings. In this regard, a number of sensitisation meetings were held to inform community members about the programme. It was at one of those meetings that the community-based Cash Transfer Committees (CTCs) were democratically established.

“We were gathered village by village and we were told to write down the names of people we wanted to get in the committee.” Tadiwa, female, caregiver benefiting from cash transfers

A key responsibility of the CTCs was to facilitate village meetings and to discuss and verify who was eligible to benefit from the programme. In this process, the wider community was involved, drawing on local experiences and knowledge. The informants commented on the importance of drawing on local knowledge, exemplified by one community member:

“They used local knowledge in selecting the deserving households. It was done well.” Raviro, male, community member

As explained earlier, CTCs were also charged with the responsibility of overseeing progress and payment, monitoring compliance of cash transfer beneficiaries who had been assigned conditions, as well as facilitating parenting skills classes. Our CTC informants described their commitment and engagement with their overseeing role:

“Our roles were to tell people two weeks before receiving their money and to see if those receiving money are supposed to receive, observe if the money is being used appropriately, also monitor if they have paid the children’s school fees and whether the children are in school, we also check if children have been vaccinated at the clinic. These were our roles and responsibilities.” Rufaro, female, CTC member

Numerous informants spoke about the importance of using local knowledge and insight to identify, target and work with vulnerable households. But the notion of ‘local’ also encompassed their close proximity, making them better at monitoring and responding to problems in a timely and apt manner.

“It helped these families because I was near them and I was familiar with their problems since we lived in the same community. If a problem arose I would let my boss know, it helped them so much.” Ndura, female, CTC member

Community meetings about the programme ensured transparency and enabled community members to take an informal role in the programme. As a result, the CTCs were not alone in monitoring how cash was spent. Community members encouraged cash transfer beneficiaries to spend their money responsibly and in a way that met the objectives of the programme.

“We don’t want to see people wasting this money. That money should be spent effectively. People would say this to those people who receive the money. This is not said with harsh words but in a nice way to encourage people to be more responsible.” Shamu, male, community member

As indicated above, cash transfer beneficiaries, village heads, elected community leaders and community members in general worked together in taking this programme forward. The above quotes exemplify how the community members endorsed the implementation process (selection, overseeing, keeping tabs) and the outcome of their involvement (responsible spending). These observations indicate how cash transfer programmes, through their very design of recognising and drawing of local resources, can be embedded into a social context. In addition to appropriating the programme to ‘the local way of doing things’, it also meant that fewer ‘outsiders’ had to be paid to help with the implementation of the programme.

### Social acceptability

Social acceptability is an important element of any development programme targeting some households and not others. Achieving fairness and overcoming jealousy was often mentioned by the informants as important, highlighting a worry that the cash transfer programme could lead to social divisions. However, it was generally agreed that the programme was fair and two programme features were highlighted as contributing to people’s judgement of the fairness of the programme. First, the process of involving available and interested community members in the verification and selection of beneficiaries was said to reduce the chances of people feeling jealous.

“It relied on the community to select beneficiaries and that helps reduce the probability of anyone feeling jealous against the beneficiaries.” Rindi, male, CTC member

“It was good. I was actually impressed to see people from my community getting organised. It showed that people just need to be given a platform to be constructive. Nobody took it personally, even if their name was called out and people would say no. Nobody really showed being offended. We all even enjoyed the exercise.” Anopa, male, CTC member

Second, the process was seen as transparent, which proved to be an important pathway to achieving fairness and community ownership.

“Because people really felt they were part of everything and they felt this was a very transparent way of doing things.” Rindi, male, CTC member

“The community liked the meetings. They showed a lot of fairness, and enhanced community ownership” Zira, male, representative from implementing agency

There was an overwhelming consensus that the programme, through community participation and transparency, had been successful in overcoming widespread jealousy and feelings of unfairness about how beneficiaries were selected. Other factors contributed to social acceptability, including a community-wide appreciation for what the programme set out to achieve: support vulnerable households.

“The whole community was happy about the programme because it developed the benefitting households in the community.” Raviro, male, community member

“You may be concerned about your neighbour’s child. You might feel pity, and want them to go to school, but cannot help financially. Then if someone comes to help the family, you become happy. ” Dova, female, caregiver benefitting from conditional cash transfers

The quotes by Raviro and Dova are indicative of the kind of empathy that characterises this rural area of Zimbabwe, and highlight more widespread benefits that go above and beyond the targeted household. Indeed, a number of informants spoke about how the programme had a positive impact on neighbours, extended family members and the community at large, taking some of the responsibility away from them to support vulnerable members of their community/family.

“People were happy because it reduced the load on their shoulders.” Daya, female, CTC member

In what ways was the programme perceived to benefit community members?

### Improved schooling and education

Although primary education is free in Zimbabwe, a growing number of schools are forced to charge pupils school development levies and tuition fees to uphold the standard of education. These fees, coupled with school-related costs such as uniforms, books, pens and paper, make primary education costly for the poorest families. As such, and with a focus on children’s education, the cash transfer programme was received positively, helping parents and guardians to cover the educational needs of their children. The cash transfers were said to have a positive impact on children’s school attendance and performance. For example, Bastirai, a 14-year-old boy who benefitted from the cash transfer programme claimed that the programme enabled both him and his siblings to pay for school development levies and tuition fees, attend school more regularly, wear new uniforms, and perform better in school. He told us that the programme had made a difference to their lives.

“We paid for my school fees and my other sibling’s school fees. We also bought some uniforms. […] Our performance has changed for the better. We used to be sent away and miss a lot of lessons. Now we are attending all lessons so things have changed for the better.” Bastirai, 14-year-old boy benefiting from cash transfers

His account is corroborated by Noah, a CTC member, who was of the impression that the programme contributed to improvements in schooling and education and that these perceived improvements were amongst the greatest achievements of the cash transfer programme.

“The most significant change is that those children who were not attending school are now attending school […] Children are now going to school looking smart.” Noah, male, CTC member

The notion of children looking smart was mentioned frequently. Children from the poorest families were, through the cash transfers, believed to visually ‘escape’ representations of poverty. There was a perception that now children were able to wear shoes, replace old and torn uniforms, and this had made them more equal to their peers and difficult to pinpoint as poor. Illustrating this ‘escape’ from poverty, one CTC member claimed that ‘now the rich and the poor are all the same’, another member said ‘the programme has brought equality to the community.’

Many guardians of households benefitting from cash transfers spoke about how they could pay the school levies and tuition fees promptly, sparing them and their children from harassment from school administrators. The ability of poor families to pay fees promptly was noticed by a school leader who felt that the programme enabled them to arrange and plan school activities better.

“There is a noticeable change. We do not see children being disturbed by being sent back home to collect their levies because they are being paid up in time plus we do not have a single drop out […] we can say most or 100% of the pupils in a class have pens and paper. So we do not have any pupil who comes to sit doing nothing or not writing… When school term started this year I noticed a difference because when we requested the levies, pupils just made the payments. So in a matter of two weeks the levies were paid up. This was unusual, we used to stretch up to end of second term talking to parents to come and pay levies. […] It made it possible for us to do some of the projects that we wanted to do, tours and visits. We do not have any arrears” Silas, male, school leader

Although this was thought to be of benefit to the school, the same school leader also said that he had observed an increase in demand for education, forcing him to send children to other schools in order to keep the student-teacher ratio acceptable.

### Improved child health and well-being

The programme was believed to have both physical and psychosocial health benefits. As a disease prevention instrument, cash transfers were believed to have enhanced vaccination rates and improved the uptake of child growth monitoring services.

“People who never used to bring their children for growth monitoring were now bringing their children for that. This is because the programme was demanding to see the child health card to check on growth monitoring and vaccinations.” Mercy, female, caregiver benefitting from conditional cash transfers

Although the cash transfer programme may have incentivised some recipients to take their children for immunisation, the programme has not had a significant effect on vaccination rates [16].

A number of examples were also given to highlight the perceived link between their increased access to money and disease prevention. Ndura, a CTC member, spoke about how the ability of poor families to now afford shoes for their children, can help prevent certain diseases contracted from soil and unhygienic floor surfaces.

“Most children now wear shoes when going to school so they are safe from the diseases found in toilets and play grounds. Also immunisation of children helped to limit diseases.” Ndura, female, CTC member

Although immunisation coverage is relatively good in Zimbabwe, the conditional cash transfer arm was believed to be particularly effective for people adhering to the Apostolic faith, whose religious beliefs prohibit them from making use of medical services. However through cash incentives and dialogue with CTC members, a number of Apostolics agreed to circumvent this rule and allowed their children to be immunised.

“Children are vaccinated in greater numbers. Before many children were not brought to the health clinic because parents said they were are in the apostolic sector. This has changed.” Zivai, female, CTC member

The cash transfers were also believed to improve the nutritional intake of children and other household members. Although the cash transfers were linked to children’s regular school attendance in the conditional cash transfer areas, it was not a requirement that households spend the actual cash transfers on school costs. If they had another source of funds for this (e.g., a relative in formal sector employment), they could continue with this arrangement. This meant that some families were in a position to divert funds and assets to improve their food intake.

“When change was left I would go and buy food so that my child eats something when going to school, I would even buy soap with the change.” Tadiwa, female, caregiver benefitting from cash transfers

“Some families have started eating healthier foods, because they could now afford cooking oil and meat here and there… some are already relying on the vegetable gardens which they started using resources from this programme. I think so much has been achieved and some people’s living standards have been raised.” Rindi, male, CTC member

The cash transfers came with a sense of security and confidence in their ability to deal with future expenditures. This meant that a number of our informants spoke about how the cash transfer programme has helped reduce levels of stress and anxiety – improving their psychosocial well-being. Bastirai explained earlier how the allocation of cash to his household managed to cover all their educational costs. This was tremendously important for him, removing worries and headaches and helping him feel more content with life.

“Last year I used to suffer from headaches because I was always thinking about my brother who was not going to school. I could not focus on my studies properly because I was troubled about my brother who will be at home and not going to school. Sometimes I would miss school and go to the bees to make some money for him to go to school. Right now I can go for 3 months without experiencing any headaches. I am now comfortable at school. I do not feel out of place.” Bastirai, 14-year-old boy benefiting from cash transfers

The psychosocial benefits of the programme were not limited to children. Also guardians expressed relief and a reduction in levels of stress as a result of having a predictable income and support in providing for their children’s education. One guardian extended this observation further by arguing that this is also a relief for the community whole, as it reduces the number of our-of-school children roaming around.

“It strengthened my family because I don’t get stressed when schools are about to open thinking about school fees. […] it has also helped the community not to worry and they are happy that I have managed to pay school fees for my children and that they will not roam around the village.” Dova, female, caregiver benefitting from conditional cash transfers

### Poverty reduction and social transformation

If cash transfers are to be considered a social protection strategy, they need to move beyond a focus on health and children’s educational gains and also consider the ways they can potentially challenge and transform the social space that leaves children vulnerable. We now report on some of the transformative opportunities that can potentially arise at a micro-level from community-led cash transfer programmes.

First of all, the programme, through its involvement with whole communities, sensitised everyone to the needs and struggles of orphaned and vulnerable children and their labour-constrained guardians. The programme was believed to ‘open eyes’ and was said to spark a sense of collective action, where groups and communities got mobilised and committed to help vulnerable children.

“I think it gave people an opportunity to look at each and every household in the community and also opened our eyes to some issues that were not given much attention, like the issue of vulnerable children. People started mobilising each other to help vulnerable children.” Anopa, male, CTC member

One of the more specific areas where community members were sensitised relates to the need to obtain birth certificates. Birth certificates are a prerequisite for any young man or women in Zimbabwe to obtain an identification document that gives them full rights as citizens. Pupils sitting their final year exams and looking to obtain a diploma need to present their birth certificate. Many health and social services in Zimbabwe require a birth certificate for their services to be made available. It is therefore crucial for children to obtain official copies of their birth certificates, but this is a bureaucratic and sometimes costly (e.g., opportunity costs related to travel) process that prevents many parents and guardians from following this through. By requiring all beneficiaries to have a birth certificate, the programme helped generate a local understanding of the importance of birth certificates.

“When the programme started, people didn’t appreciate the importance of birth certificates to children and their personal national identity cards. The programme took time to explain the importance of these papers. Everyone took this seriously and made an effort to process their papers. People who didn’t have money to process the documents were given the money. I believe that even schools now will not have problems of pupils without documentation as most parents have made an effort to get these documents. After this programme, every pupil should be able to write their grade seven exams because they will all have their birth certificates.” Kokayi, male, CTC member

Second, informants argued that the programme, through its provision of cash, distinguished itself from other programmes by giving people a sense of control over their lives. It allowed people to prioritise their own needs rather than having their needs prescribed by non-governmental organisations. Through this sense of control, families were said to be able to transform their lives, with many families using their cash grants to start income generating projects.

“I learned that having paid for school fees we should use the remaining bit of money to buy seedlings and do gardening so that when the program goes you will not be left out with nothing at all.” Tadiwa, female, caregiver benefitting from cash transfers

Not everyone had enough spare money to buy farming implements. But with the programme also came a rise in informal savings and lending groups, where cash transfer beneficiaries, used their steady income as a guarantor to join a local savings and lending group to set up an income generating activity. As highlighted by Tadiwa, many people felt that this was important exit strategy of the programme. This rise in income generating activities was also noted by Shamu, a community member not benefitting from the programme.

“Definitely there has been a change because a lot of people are now involved in a lot of small projects. For example, at this centre, a lot of people are now involved in small projects. A lot of women are involved in buying and selling of clothes and milk. Some are selling tomatoes. So at least people are engaged in some income generating project. People are now making money instead of just waiting for donations. People have changed their behaviour; they are now very seriously looking for money.” Shamu, male, community member

Shamu highlights how the cash transfer programme in a rather paradoxical way has changed people’s behaviour, with recipients being focused on generating income themselves, disassociating themselves from the idea that they may be passive recipients of aid.

Third, the programme, as discussed earlier, brought a sense of social equality into the communities. By enabling children to pay for their school fees in time and avoid being sent home, they and their families avoid being ostracised as poor and vulnerable, which, according to a school leader, can have a transformative impact on children’s lives.

“Cash transfer is very effective, I wish it would continue operating, and there won’t be any difference between our children such that we will not be able to see the children who would have come from poor families. It can be very embarrassing for a child to be labelled as poor because they did not pay for their school fees. It also exposes the whole family. Such public humiliation is not good because it can make the child not reach his/her self-esteem.” Silas, male, school leader

A community member not benefiting from the programme also noted the change that had happened with people being more equal. Possibly reflecting the increased awareness of the needs of struggling families and an enhanced social solidarity in the community, she argues that the community has become more unified, with everyone interacting well with each other across social strata.

“It brought more social cohesion because some people used to suffer on their own. They did not socialize with other people because they were poor but with the coming of the programme everyone is working together, people are now interacting with everyone.” Florence, female, community member

The subjective experiences reported on in this section suggest that people felt the programme facilitated social transformation at a local level through 1) a sensitisation and mobilisation of community members on the needs of vulnerable children; 2) individual control and income generating activities; and 3) by making community members more equal.

### Persisting social and logistical challenges

Whilst community members generally spoke highly about the programme, there were persisting social and logistically challenges. For example, there were some accounts of beneficiaries feeling that they were no longer greeted by some people in the community and attributed this to jealousy. Some people said that there were community members feeling disgruntled and left out, failing to understand why they have not been supported when their equally poor neighbour has. This however was not surprising to the informants.

“It is common for people to be jealous. When you are getting something and they are not, it will compromise the cohesion of the community; they will be jealous and question why they were left out?” Anashe, female, caregiver benefitting from cash transfers

“Where there are people there will always be jealousy. There are people who are naturally jealous and there are people who are not jealous. I however have never heard of people who have said they are jealous. People believe that the people who were selected were the deserving households.” Raviro, male, community member

So whilst the programme did not completely eradicate jealousy, informants spoke about how feelings of jealousy changed over time, arguing that people eventually got used to the idea that some community members, and not others, received money from an external organisation.

“Jealousy was there in the beginning but now people seem to be getting along well with each other.” Ruko, male, community member

Adding to the phenomenon of jealousy was the question of targeting. A number of examples emerged from the interviews where people expressed the concern that the targeting and selection process had bypassed deserving households, and included households that perhaps were not as deserving.

“There are families we feel they should have been included, for example, there is a family with a very old women, who spends the whole day in the garden and is surviving on selling vegetables, but she has 3–4 orphans” Tongo, female, caregiver benefitting from cash transfers

We have discussed the challenges of targeting elsewhere [27]. Logistical challenges also deserve mentioning, both to give us an insight to some of the challenges that the CTCs were confronted with and to enable future programme planners to recognise and overcome these challenges. At the household level, illness and disability, as well as dysfunctional family dynamics presented difficulties to reaching some of the most vulnerable children. 14-year-old Bastirai gives an example of how his sick father had struggled to pick up the cash grants from the pay-point. He also spoke about how the CTCs responded to the difficulties experienced by his dad by eventually allowing a representative to go and collect the money on his behalf.

“My father used to go and collect the money but after collecting the money he would come back and complain that his leg was in pain. He would spend five days sleeping after that. Those days the people would say you cannot send representatives to get the money on your behalf.” Bastirai, 14-year-old boy benefiting from cash transfers

Another frequently mentioned problem expressed by our informants was the irresponsible use of cash grants by men. Men were often reported as unsympathetic to the needs of children in their care and more interested in their own personal needs. One male CTC member brought this up in a focus group discussion with other CTC members who agreed to challenge.

“There are some men in the community who had difficulties in understanding the programme. These men do not work they just stay at home. So when the money came they took the money used it to meet their own needs instead of using it for the benefit of their children. Instead of paying for school fees and buying uniforms, the men went drinking and paid off their own debts.” Kokayi, male, CTC member

CTC members also had to respond to changes in household composition. Divorce and migration meant that some families were split up, leaving parents, or indeed other caregivers, to fight for the child, partially motivated by the cash grants.

“In that in a family perhaps it might cause discontent in a household like where I almost observed where a mother would have been divorced from the father and she gets the money while staying away from the father it caused a lot of problems because the father wants the money and the mother wants the money so they will be fighting for the child even others who are not fathers, grandmothers and other relatives if they know that that family is getting money through the child they will start fighting for the child. There are quite a number of such cases.” Kuda, male, representative from implementing agency

Some CTC members had to ensure compliance to conditions in the conditional cash transfer communities. This included encouraging parents to take their children for vaccinations. However, Apostolic parents refused to take their children to the hospital on religious grounds, requiring CTC members to act as their compliance buddies and engage in a dialogue encouraging them to take their child for vaccination. Sometimes they were successful, other times not.

“There were people in the Apostolic sect who were adamant saying we are not allowed by our religion to our children for vaccinations. We would engage them in a discussion and, in the end, you see the child being taken for vaccinations.” Dzingai, male, CTC member

It is clear that social divisiveness cannot be completely overcome and that many logistical issues, some unavoidable (e.g., divorce and family break-ups), still present significant challenges to the implementation of cash transfer programmes.

## Discussion

This paper has examined local perceptions of a community-led cash transfer programme in eastern Zimbabwe. More specifically, the paper explored their experiences of participating in the programme and its impact on children and the communities at large. Community participation was said to ensure the programme resonated with local knowledge systems, norms and structures. Community members, through village meetings and community-based cash transfer committees, felt they were given the opportunity to draw on local knowledge and resources to select, support and monitor cash transfer beneficiaries. Community members thus respected the programme. They recognised that the way the programme was implemented made the selection process fair and transparent – enabling collective ownership of the programme and limiting, not eradicating, social divisiveness. They were sympathetic to what the programme sought to do and recognised the different ways the programme benefitted poor households. For example, people felt that the greatest impact of the programme pertained to improvements in children’s school attendance and performance. Prompt payment of school fees and children being given new school uniforms meant that children were not sent home from school, allowing them to concentrate on their studies. The programme was also perceived to have had noteworthy influences on the physical and psychosocial health of children. People were of the belief that the increase in income meant that children had access to more nutritious food. More children were also said to be taken for vaccinations and growth monitoring at the local health clinic – although the measured effect of cash transfers on vaccination uptake was not statistically significant [16]. The programme was also said to come as a relief for children and their guardians, removing worries and reducing levels of stress. These benefits have also been noted by cash transfer beneficiaries in Malawi [30]. The programme was also believed to provide beneficiaries and the communities with opportunities for social transformation. At a household-level, although this may not be particular to community-led cash transfer programmes, some guardians spoke of how the programme had enabled them to set up small-scale income generating activities, transforming their livelihoods by strengthening household assets. Moreover, social sanctions arising from the transparency and involvement of community members were observed to encourage household recipients to take an active role in distancing themselves from being passive recipients of aid, to agents of change who work for a brighter future of their children. At a community-level, the programme was said to sensitise community members to the needs of orphaned and vulnerable children and fostered a sense of collective action for programme success. The programme was said to address social inequalities – creating more unified and socially cohesive community contexts.

There were also examples of the limitations and complexity of involving communities. Communities are made up of complex webs of power relations and interests that result in some people, often the most vulnerable, being excluded and ostracised. Jealousy was therefore not eradicated and we heard of examples of how some undeserving households were included in the beneficiary list at the expense of more deserving households. There was also an acknowledgement of how some of the local structures and dynamics may have had a negative impact on the programme, such as the role of religion (in this case Apostolics), dysfunctional family relations, illness and disability, and the conflicting priorities of some men.

This paper does not seek to draw causal relationships. For example, we cannot say for certain that community participation led to social acceptability. However, the perspectives presented in this exploratory study do suggest that such a link may indeed be possible. In contrasts with observations of social divisiveness from other cash transfer initiatives in sub-Saharan Africa e.g., [17,18], the programme we report on appears to have had some level of buy-in from community members, particularly from those who participated in this study. Relatedly, people felt generally happy about the programme, expressing their positive impressions of the impact that the programme had on beneficiary households and the community at large. More research however is needed to determine factors and prerequisites for social acceptability.

Adato and Bassett [14] in their asset-based social protection framework reduce the role of cash transfers to protective (secure basic needs) and preventative (avert asset reduction) effects. This rather limited role of cash transfers has sparked debate on how cash transfers can be more transformative [e.g., [31,32]. Our experiences suggest that cash transfers, if directed by community members, can, using the same language as the framework proposed by Adato and Bassett (2009), be promotional (enable people to save and accumulate assets) and transformational (create supportive social environments). Similar observations have been made in Kenya through community-based capital cash transfers [33,34].

A couple of key limitations deserve mentioning. First, we were not in a position to draw links between personal accounts and their unique context. A limitation of this paper is therefore that it does not present a fine-grained analysis of differences between communities receiving conditional or unconditional cash transfers, or whether the communities are located in roadside settlements, near forestry plantations or in subsistence farming areas. Second, our findings suggest an overwhelmingly positive attitude towards the programme. Whilst this may be the case, there is also the risk that there could be some reporting bias in the paper, with participants, in the hope of continued support, deliberately communicating positive aspects of the programme.

## Conclusions

Notwithstanding these limitations, we conclude that community-led cash transfer initiatives have the potential to open up possibilities for community participation and agency, and thereby can be more aligned with local realities, that make it possible to achieve social acceptability, facilitate more transformational change, and enable people, both those who benefit directly and those who do not, to see the benefits of the programme.

## Competing interests

The authors declare that they have no competing interests.

## Authors' contributions

MS managed the data set, performed the data analysis, and drafted the manuscript. PM organised the field team that collected and transcribed the data and conducted some of the interviews. LR designed the overarching cash transfer trial and participated in the design of this sub-study. SM participated in the design and implementation of the programme. LS participated in the design of the study. CN managed the research and supervised data collection. SG conceived and oversaw the development and conduct of the cash transfer trial including this sub-study. All authors contributed to the final write-up and have read and approved the final manuscript.

## Authors' information

At the time of the study, MS was with the Department of Health Promotion and Development, University of Bergen, Bergen, Norway. He is now with Save the Children UK, London, UK. PM and SM are with the Biomedical Research and Training Institute (BRTI), Harare, Zimbabwe. LR is with the Department of Infectious Disease Epidemiology, Imperial College, London, UK. LS is with the Department of Infection and Population Health, University College London, London, UK. CN and SG are with the BRTI and the Department of Infectious Disease Epidemiology, Imperial College, London, UK.

## Pre-publication history

The pre-publication history for this paper can be accessed here:

http://www.biomedcentral.com/1471-2458/13/342/prepub
